# Challenges to implementing artificial intelligence in healthcare: a qualitative interview study with healthcare leaders in Sweden

**DOI:** 10.1186/s12913-022-08215-8

**Published:** 2022-07-01

**Authors:** Lena Petersson, Ingrid Larsson, Jens M. Nygren, Per Nilsen, Margit Neher, Julie E. Reed, Daniel Tyskbo, Petra Svedberg

**Affiliations:** 1 School of Health and Welfare, Halmstad University, Box 823, 301 18 Halmstad, Sweden; 2 Department of Health, Medicine and Caring Sciences, Division of Public Health, Faculty of Health Sciences, Linköping University, Linköping, Sweden; 3 Department of Rehabilitation, School of Health Sciences, Jönköping University, Jönköping, Sweden

**Keywords:** Artificial intelligence, Digital transformation, Healthcare, Implementation, Healthcare leaders, Organizational change, Qualitative methods, Stakeholders

## Abstract

**Background:**

Artificial intelligence (AI) for healthcare presents potential solutions to some of the challenges faced by health systems around the world. However, it is well established in implementation and innovation research that novel technologies are often resisted by healthcare leaders, which contributes to their slow and variable uptake. Although research on various stakeholders’ perspectives on AI implementation has been undertaken, very few studies have investigated leaders’ perspectives on the issue of AI implementation in healthcare. It is essential to understand the perspectives of healthcare leaders, because they have a key role in the implementation process of new technologies in healthcare. The aim of this study was to explore challenges perceived by leaders in a regional Swedish healthcare setting concerning the implementation of AI in healthcare.

**Methods:**

The study takes an explorative qualitative approach. Individual, semi-structured interviews were conducted from October 2020 to May 2021 with 26 healthcare leaders. The analysis was performed using qualitative content analysis, with an inductive approach.

**Results:**

The analysis yielded three categories, representing three types of challenge perceived to be linked with the implementation of AI in healthcare: 1) Conditions external to the healthcare system; 2) Capacity for strategic change management; 3) Transformation of healthcare professions and healthcare practice.

**Conclusions:**

In conclusion, healthcare leaders highlighted several implementation challenges in relation to AI within and beyond the healthcare system in general and their organisations in particular. The challenges comprised conditions external to the healthcare system, internal capacity for strategic change management, along with transformation of healthcare professions and healthcare practice. The results point to the need to develop implementation strategies across healthcare organisations to address challenges to AI-specific capacity building. Laws and policies are needed to regulate the design and execution of effective AI implementation strategies. There is a need to invest time and resources in implementation processes, with collaboration across healthcare, county councils, and industry partnerships.

## Background

The use of artificial intelligence (AI) in healthcare can potentially enable solutions to some of the challenges faced by healthcare systems around the world [1–3]. AI generally refers to a computerized system (hardware or software) that is equipped with the capacity to perform tasks or reasoning processes that we usually associate with the intelligence level of a human being [4]. AI is thus not one single type of technology but rather many different types within various application areas, e.g., diagnosis and treatment, patient engagement and adherence, and administrative activities [5, 6]. However, when implementing AI technology in practice, certain problems and challenges may require an optimization of the method in combination with the specific setting. We may therefore define AI as complex sociotechnical interventions as their success in a clinical healthcare setting depends on more than the technical performance [7]. Research suggests that AI technology may be able to improve the treatment of many health conditions, provide information to support decision-making, minimize medical errors and optimize care processes, make healthcare more accessible, provide better patient experiences and care outcomes as well as reduce the per capita costs of healthcare [8–10]. Even if the expectations for AI in healthcare are great [2], the potential of its use in healthcare is far from having been realized [5, 11, 12].

Most of the research on AI in healthcare focuses heavily on the development, validation, and evaluation of advanced analytical techniques, and the most significant clinical specialties for this are oncology, neurology, and cardiology [2, 3, 11, 13, 14]. There is, however, a current research gap between the development of robust algorithms and the implementation of AI systems in healthcare practice. The conclusion in newly published reviews addressing regulation, privacy and legal aspects [15, 16], ethics [16–18], clinical and patient outcomes [19–21] and economic impact [22], is that further research is needed in a real-world clinical setting although the clinical implementation of AI technology is still at an early stage. There are no studies describing implementation frameworks or models that could inform us concerning the role of barriers and facilitators in the implementation process and relevant implementation strategies of AI technology [23]. This illustrates a significant knowledge gap on how to implement AI in healthcare practice and how to understand the variation of acceptance of this technology among healthcare leaders, healthcare professionals, and patients [14]. It is well established in implementation and innovation research that novel technologies, such as AI, are often resisted by healthcare leaders, which contributes to their slow and variable uptake [13, 24–26]. New technologies often fail to be implemented and embedded in practice because healthcare leaders do not consider how they fit with or impact existing healthcare work practices and processes [27]. Although, understanding how AI technologies should be implemented in healthcare practice is unexplored.

Based on literature from other scientific fields, we know that the leaders’interest and commitment is widely recognized as an important factor for successful implementation of new innovations and interventions [28, 29]. The implementation of AI in healthcare is thus supposed to require leaders who understand the state of various AI systems. The leaders have to drive and support the introduction of AI systems, the integration into existing or altered work routines and processes, and how AI systems can be deployed to improve efficiency, safety, and access to healthcare services [30, 31]. There is convincing evidence from outside the healthcare field of the importance of leadership for organizational culture and performance [32], the implementation of planned organizational change [33], and the implementation and stimulation of organizational innovation [34]. The relevance of leadership to implementing new practices in healthcare is reflected in many of the theories, frameworks, and models used in implementation research that analyses barriers to and facilitators of its implementation [35]. For example, Promoting Action on Research Implementation in Health Services [36], Consolidated Framework for Implementation Research (CFIR) [37], Active Implementation Frameworks [38], and Tailored Implementation for Chronic Diseases [39] all refer to leadership as a determinant of successful implementation. Although these implementation models are available and frequently used in healthcare research, they are highly abstract and not tailored to the implementation of AI systems in healthcare practices. We thus do not know if these models are applicable to AI as a socio-technical system or if other determinants are important for the implementation process. Likewise, based on a new literature study, we found no AI-specific implementation theories, frameworks, or models that could provide guidance for how leaders could facilitate the implementation and realize the potential of AI in healthcare [23]. We thus need to understand what the unique challenges are when implementing AI in healthcare practices.

Research on various types of stakeholder perspectives on AI implementation in healthcare has been undertaken, including studies involving professionals [40–43], patients [44], and industry partners [42]. However, very few studies have investigated the perspectives of healthcare leaders. This is a major shortcoming, given that healthcare leaders are expected to have a key role in the implementation and use of AI for the development of healthcare. Petitgand et al.’s study [45] serves as a notable exception. They interviewed healthcare managers, providers, and organizational developers to identify barriers to integrating an AI decision-support system to enhance diagnostic procedures in emergency care. However, the study did not focus on the leaders’ perspectives, and the study was limited to one particular type of AI solution in one specific care department. Our present study extends beyond any specific technology and encompasses the whole socio-technical system around AI technology. The present study thus aimed to explore challenges perceived by leaders in a regional Swedish healthcare setting regarding implementation of AI systems in healthcare.

## Methods

### Design

This study took an explorative qualitative approach to understanding healthcare leaders’ perceptions in contexts in which AI will be developed and implemented. The knowledge generated from this study will inform the development of strategies to support an AI implementation and help avoid potential barriers. The analysis was based on qualitative content analysis, with an inductive approach [46]. Qualitative content analysis is widely used in healthcare research [46] to find similarities and differences in the data, in order to understand human experiences [47]. To ensure trustworthiness, the study is reported in accordance with the Consolidated Criteria for Reporting Qualitative Research 32‐item checklist [48].

### Setting

The study was conducted in a county council (also known as “region”) in the south of Sweden. The Swedish healthcare system is publicly financed based on local taxation; residents are insured by the state and there is a vision that healthcare should be equally accessible across the population. Healthcare responsibility is decentralized to 21 county councils, whose responsibilities include healthcare provision and promotion of good health for citizens.

The county council under investigation has since 2016 invested financial, personnel and service resources to enable agile analysis (based on machine learning models) of clinical and administrative data of patients in healthcare [49, 50]. The ambition is to gain more value from the data, utilizing insights drawn from machine learning on healthcare data to make facts-based decisions on how healthcare is managed, organized, and structured in routines and processes. The focus is thus on overall issues around management, staffing, planning and standardization for optimization of resource use, workflows, patient trajectories and quality improvement at system level. This includes several layers within the socio-technical ecosystem around the technology, dealing with: a) generating, cleaning, and labeling data, b) developing models, verifying, assuring, and auditing AI tools and algorithms, c) incorporating AI outputs into clinical decisions and resource allocation, and d) the shaping of new organizational structures, roles, and practices. Given that AI thus extends beyond any specific technology and encompasses the whole socio-technical system around the technology, in the context of this article, it is hereafter referred to generically as ‘AI systems’. We deliberately sought to understand the broad perspectives on healthcare leaders in a region that has a high level of support for AI developments and our study thus focuses on the potential of a wide range of AI systems that could emerge from the regional investments, rather than a specific AI application or AI algorithms.

### Participants

Given the focus on understanding healthcare leaders’ perceptions, we purposively recruited leaders who were in a position to potentially influence the implementation and use of AI systems in relation to the setting described above. To achieve potential variability, these leaders belonged to three groups: politicians at the highest county council level, managers at various levels, such as the hospital director, manager for primary care, manager for knowledge and evidence, head of research and development center, and quality developers and strategists with responsibilities for strategy-based work at county council level or development work in various divisions in the county council healthcare organization.

The ambition was to include leaders who had a range of experiences, interests and with different mandates and responsibilities in relation to funding, running, and sustaining the implementation of AI systems in practice. A sample of 28 healthcare leaders was invited through snowball recruitment; two declined and 26 agreed to participate (Table 1). This sample comprised five individuals originally identified on the basis of their knowledge and insights. They were interviewed and they then identified and suggested other leaders to interview.

**Table 1:** Participants’ characteristics (*n* = 26)

**Role**	
Politicians	4
Managers	9
Quality developers and strategists	13
**Context**	
Healthcare administration	15
Primary care	6
Psychiatry	3
Hospital	2
**Gender**	
Male	18
Female	8

### Data collection

Individual semi-structured interviews were conducted between October 2020 and May 2021 via phone or video communication by one of the authors (LP or DT). We start from a broad perspective on AI focusing on healthcare leaders’ perceptions bottom-up and not on the views of AI experts or healthcare professionals who work with specific AI algortihms in clinical practice. The interviews were based on an interview guide, structured around: 1) the roles and previous experiences of the informants regarding the application of AI systems in practice, 2) the opportunities and problems that need to be considered to support implementation of AI systems, 3) beliefs and attitudes towards the possibilities of using AI systems to support healthcare improvements, and 4) the obstacles, opportunities and facilitating factors that need to be considered to enable AI systems to fit into existing processes, methods and systems. The interview guide was thus based on important factors previously identified in terms of implementing technology in healthcare [51, 52]. Interviews lasted between 30 and 120 min, with a total length of 23 h and 49 min and were audio-recorded.

### Data analysis

An inductive qualitative content analysis [46] was used to analyze the data. First, the interviews were transcribed verbatim and read several times by the first (LP) and second (IL) authors, to gain familiarity. Then, the first (LP) and second (IL) authors conducted the initial analyses of the interviews, by identifying and extracting meaning units and/or phrases with information relevant to the object of the study. The meaning units were then abstracted into codes, subcategories, and categories. The analytical process was discussed continuously between authors (LP, IL, JMN, PN, MN, PS). Finally, all authors, who are from different disciplines, reviewed and discussed the analysis to increase the trustworthiness and rigour of the analysis. To further strengthen the trustworthiness, the leaders’ quotations used in this paper were translated from Swedish to English by a native English-speaking professional proofreader and were edited only slightly to improve readability.

## Results

**Fig. 1:**
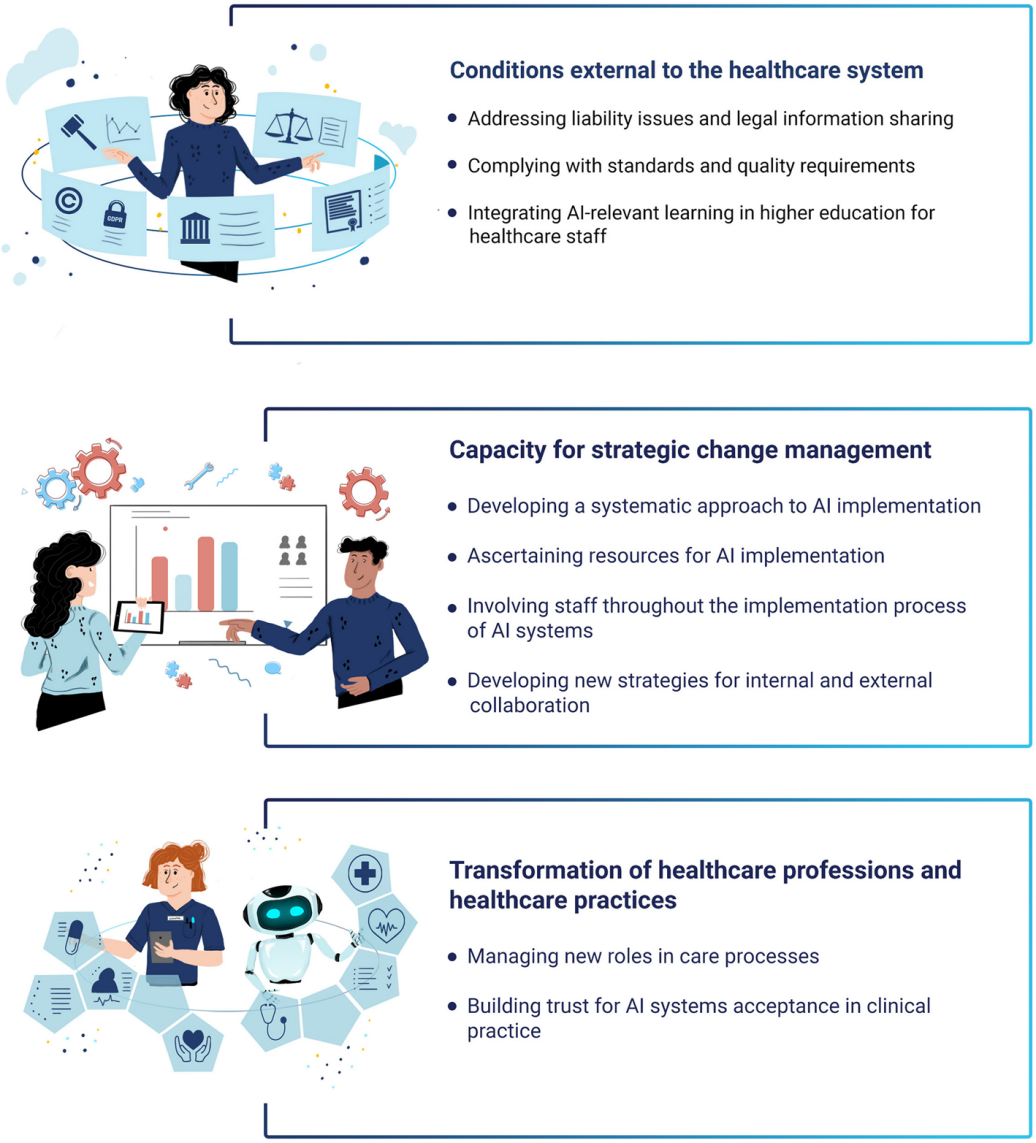
Categories and subcategories

Three categories consisting of nine sub-categories emerged from the analysis of the interviews with the healthcare leaders (Fig. 1). *Conditions external to the healthcare system* concern various exogenous conditions and circumstances beyond the direct control of the healthcare system that the leaders believed could affect AI implementation. *Capacity for strategic change management* reflects endogenous influences and internal requirements related to the healthcare system that the leaders suggested could pose challenges to AI implementation. *Transformation of healthcare professions and healthcare practice* concerns challenges to AI implementation observed by the leaders, in terms of how AI might change professional roles and relations and its impact on existing work practices and routines.

### Conditions external to the healthcare system

#### Addressing liability issues and legal information sharing

The healthcare leaders described the management of existing laws and policies for the implementation of AI systems in healthcare as a challenge and an issue that was essential to address. According to them, the existing laws and policies have not kept pace with technological developments and the organization of healthcare in today’s society and need to be revised to ensure liability.

The accountability held among individuals, organizations, and AI systems regarding decisions based on support from an AI algorithm was perceived as a risk and an element that needs to be addressed. However, accountability is not addressed in existing laws, which were perceived by the leaders to present problematic uncertainties in terms of responsibilities. They raised concerns about where responsibilities lie in relation to decisions made by AI algorithms, such as when an AI algorithm run in one part of the system identifies actions that should be taken in another part of the system. For example, if a patient is given AI-based advice from a county council-operated patient portal for triaging suggesting self-care, and the advice instead should have been to visit the emergency department, who has the responsibility, is it the AI system itself, the developers of the system or the county council. Additionally, concerns were raised about accountability, if it turns out that the advice was not accurate.

*The issue of accountability is a very difficult one. If I agree with what doctor John (AI systems) recommended, where does the burden of proof lie? I may have looked at this advice and thought that it worked quite well. I chose to follow this advice, but can I blame Doctor John? The legislation is a risk that we have to deal with.* Leader 7.

Concerns were raised as to how errors would be handled when AI systems contributed to decision making, highlighting the need for clear laws and policies. The leaders emphasized that, if healthcare professionals made erroneous decisions based on AI systems, they could be reported to the Patients Advisory Committee or have their medical license revoked. This impending threat could lead to a stressful situation for healthcare professionals. The leaders expressed major concerns about whether AI systems would be support systems for healthcare professionals’ decisions or systems that could take automated and independent decisions. They believed based on the latter interpretation that there would be a need for changes in the laws before they could be implemented in practice. Nevertheless, some leaders anticipated a development where some aspects of care could be provided without any human involvement.

*If the legislation is changed so that the management information can be automated, that is to say that they start acting themselves, but they’re not allowed to do that yet. It could, however, be so that you open an app in a few years’ time, then you furnish the app with the information that it needs about your health status. Then the app can write a prescription for medication for you, because it has all the information that is needed. That is not allowed at present, because the judicial authority still need an individual to blame when something goes wrong. But even that aspect will be gradually developed.* Leader 2.

According to the leaders, legislation and policies also constituted obstacles to the foundation in the implementation of AI systems in healthcare: collecting, using, merging, and analyzing patient information. The limited opportunities to legally access and share information about patients within and between organizations were described as a crucial obstacle in implementing and using AI systems. Another issue was the legal problems when a care provider wanted to merge information about patients from different providers, such as the county council and a municipality. For this to take place, it was perceived that a considerable change of the laws regulating the possibilities of sharing information across different care providers would be required. Additionally, there are challenges in the definition of personal data in laws regulating personal integrity and in the risk of individuals being identified when the data is used for computerized advanced analytics. The law states that it is not legal to share personal data, but the boundaries of what is constituted by personal data in today’s society are changing, due to the increasing amounts of data and opportunities for complex and intelligent analysis.

*You are not allowed to share any personal information. No, we understand that but what is personal information and when is personal information no longer personal information? Because legally speaking it is definitely not just the case of removing the personal identity number and the name, as a computer can still identify who you are at an individual level. When can it not do that?* Leader 2.

Thus, according to the healthcare leaders, laws and regulations presented challenges for an organization that want to implement AI systems in healthcare practice, as laws and regulations have different purposes and oppose each other, e.g., the Health and Medical Services Act, the Patient Act and the Secrecy Act. Leaders described how outdated laws and regulations are handled in healthcare practice, by stretching current regulations and attempts to contribute to changing laws*.* They aimed to not give up on visions and ideas, but to try to find gaps in existing laws and to use rather than break the laws. When possible, another way to approach this was to try to influence decision-makers on the national political level to change the laws. The leaders reported that civil servants and politicians in the county council do this lobbying work in different contexts, such as the parliament or the Swedish Association of Local Authorities and Regions (SALAR).

*We discuss this regularly with our members of parliament with the aim of influencing the legislative work towards an enabling of the flow of information over boundaries. It’s all a bit old-fashioned.* Leader 16.

#### Complying with standards and quality requirements

The healthcare leaders believed it could be challenging to follow standardized care processes when AI systems are implemented in healthcare. Standardized care processes are an essential feature that has contributed to development and improved quality in Swedish healthcare. However, some leaders expressed that the implementation of AI systems could be problematic because of uncertainties regarding when an AI algorithm is valid enough to be a part of a standardized care process. They were uncertain about which guarantees would be required for a product or service before it would be considered “good enough” and safe to use in routine care. An important legal aspect for AI implementation is the updated EU regulation for medical devices (MDR) that came into force in May 2021. According to one of the leaders, this regulation could be problematic for small innovative companies, as they are not used to these demands and will not always have the resources needed to live up to the requirements. Therefore, the leaders perceived that the county council should support AI companies to navigate these demands, if they are to succeed in bringing their products or services to implementation in standardized care processes.

*We have to probably help the narrow, supersmart and valuable ideas to be realized, so that there won’t be a cemetery of ideas with things that could have been good for our patients, if only the companies had been given the conditions and support to live up to the demands that the healthcare services have and must have in terms of quality and security.* Leader 2.

#### Integrating AI-relevant learning in higher education for healthcare staff

The healthcare leaders described that changes needed to be made in professional training, so that new healthcare professionals would be prepared to use digital technology in their practical work. Some leaders were worried that basic level education for healthcare professionals, such as physicians, nurses, and assistant nurses has too little focus on digital technology in general, and AI systems in particular. They stated that it is crucial that these educational programs are restructured and adapted to prepare students for the ongoing digitalization of the healthcare sector. Otherwise, recently graduated healthcare professionals will not be ready to take part in utilizing and implementing new AI systems in practice.

*I am fundamentally quite concerned that our education, mainly when it comes to the healthcare services. Both for doctors and nurses and also assistant nurses for that matter. That it isn’t sufficiently proactive and prepare those who educate themselves for what will come in the future. // I can feel a certain concern for the fact that our educations do not actually sufficiently prepare our future co-workers for what everybody is talking now about that will take place in the healthcare services.* Leader 15.

### Capacity for strategic change management

#### Developing a systematic approach to AI implementation

The healthcare leaders described that there is a need for a systematic approach and shared plans and strategies at the county council level, in order to meet the challenge of implementing AI systems in practice. They recognized that it will not be successful if the change is built on individual interests, instead of organizational perspectives. According to the leaders, the county council has focused on building the technical infrastructure that enables the use of AI algorithms. The county council have tried to establish a way of working with multi-professional teams around each application area for AI-based analysis. However, the leaders expressed that it is necessary to look beyond the technology development and plan for the implementation at a much earlier stage in the development process. They believed that their organization generally underestimated the challenges of implementation in practice. Therefore, the leaders believed that it was essential that the politicians and the highest leadership in the county council both support and prioritize the change process. This requires an infrastructure for strategic change management together with clear leadership that has the mandate and the power to prioritize and support both development of AI systems and implementation in practice. This is critical for strategic change to be successful.

*If the County Council management does not believe in this, then nothing will come of it either, the County Council management have to indicate in some way that this is a prioritized issue. It is this we are going to work with, then it’s not sufficient for a single executive director who pursues this and who thinks it’s interesting. It has to start at the top and then filter right through, but then the politicians have to also believe in this and think that it’s important.* Leader 4.

Additionally, the healthcare leaders experienced that there was increasing interest among unit managers within the organization in using data for AI-based analysis and that there might be a need to make more prioritizations of requests for data analysis in the future. The leaders expressed that it would not be enough to simply have a shared core facility supporting this. Instead, management at all levels should also be involved and active in prioritization, based on their needs. They also perceived that the implementation of AI systems will demand skilled and structured change management that can prioritize and that is open to new types of leadership and decision-making processes. Support for innovative work will be needed, but also caution so that change does not proceed too quickly and is sufficiently anchored among the staff. The implementation of AI systems in healthcare was anticipated to challenge old routines and replace them with new ones, and that, as a result, would meet resistance from the staff. Therefore, a prepared plan at the county council level was perceived to be required for the purpose of “anchoring” with managers at the unit level, so that the overall strategy would be aligned with the needs and views of those who would have to implement it and supported by the knowledge needed to lead the implementation work.

*It’s in the process of establishing legitimacy that we have often erred, where we’ve made mistakes and mistakes and mistakes all the time, I’ve said. That we’re not at the right level to make the decisions and that we don’t follow up and see that they understand what it’s about and take it in. It’s from the lowest manager to the middle manager to executive directors to politicians, the decisions have to have been gained legitimacy otherwise we’ll not get the impetus.* Leader 21.

The leaders believed that it was essential to consider how to evaluate different parts of the implementation process. They expressed that method development is required within the county council, because, at the moment, there is a lack of knowledge and guidelines on how to evidence-base the use of AI systems in practice. There will be a need for a support organization spanning different levels within the county council, to guide and supervise units in the systematic evaluation of AI implementations. There will also be a need for quantitative evaluation of the clinical and organizational effects and qualitative assessment that focuses on how healthcare professionals and patients experience the implementation. Additionally, validation and evaluation of AI algorithms will be needed, both before they can be used in routine care, and afterwards, to provide evidence of quality improvements and optimizations of resources.

*I believe that one needs to get an approval in some way, perhaps not from the Swedish Medical Products Agency, but the AI Agency or something similar. I don’t know. The Swedish National Board of Health and Welfare or some agency needs to go in and check that it is a sufficiently good foundation that they have based this algorithm on. So that it can be approved for clinical use.* Leader 10.

Furthermore, the leaders described a challenge around how the implementation of AI systems in practice could be sustainable and last over time. They expressed that the county council should develop strategies in the organization so that they are readied for sustainability and long-term implementation. At the same time, this is an area with fast development and high uncertainty about the future, and thus what AI systems and services will look like in five or ten years, and how healthcare professionals and patients will use them. This is a challenge and requires that both leaders and staff are prepared to adjust and change their ways of working during the implementation process, including continuous improvements and uptake, updating and evolution of technologies and work practices.

*The rate of change where digitalization, technology, new technology and AI is concerned is so high and the rate of implementation is low, so this will entail that as soon as we are about to implement something then there is something else in the market that is better. So I think it’s important to dare to implement something that is a little further on in the future.* Leader 13.

#### Ascertaining resources for AI implementation

The leaders emphasized the importance of training for implementation of AI systems in healthcare. The county council should provide customized training at the workplace and extra knowledge support for certain professions. This could result in difficult decisions regarding what and whom to prioritize. The leaders discussed whether there was a need to provide all staff with basic training on AI systems or if it would be enough to train some of them, such as quality developers, and provide targeted training for some healthcare professionals who are close to the implementation of the AI system at a care unit. Furthermore, the leaders described that the training had to be connected to implementing the AI system at a specific care unit, which could present a challenge for the planning and realization. They emphasized that it could be a waste of resources to educate the staff beforehand. They need to be educated in close connection to the implementation of a specific AI system in their workplace, which thus demands organizational resources and planning.

*I think that we often make the mistake of educating first, and then you have to use it. But you have been educated, so now you should know this? Yes, but it is not until we use something that the questions arise.* Leader 13.

There could also be a need for patient education and patient guidance, if they are to use AI systems for self-care or remote monitoring. Thus, it is vital to give all citizens the same opportunities to access and utilize new technical solutions in healthcare.

*We treat all our patients equally now, everyone will receive the same invitation, and everyone will need to ring about their appointment, although 99% could really book and do this themselves. Then we should focus on that, and thus return the impetus and the power to the patient and the population for them to take care of this themselves to a greater extent. But then of course information is needed and that in turn needs intuitive systems. That is not something we are known for.* Leader 14.

Many of the healthcare leaders found financial resources and time, especially the prioritization of time, to be critical to the implementation process of AI system. There is already time pressure in many care units, and it can be challenging to set aside time and other resources for the implementation.

#### Involving staff throughout the implementation process of AI systems

The healthcare leaders stated that anchoring and involving staff and citizens is crucial to the successfully implementation of AI systems. The management has to be responsible for the implementation process but also ensure that the staff are aware of and interested in the implementation, based on their needs. Involvement of the staff together with representatives from patient groups was considered key to successful implementation and to limit risks of perceiving the AI system as unnecessary and erroneously used. At the same time, the leaders described that it would be important for unit managers to “stand up” for the change that is required, if their staff questioned the implementation.

*I think for example that if you’re going to make a successful implementation then you have to perhaps involve the co-workers. You can’t involve all of them, but a representative sample of co-workers and patients and the population who are part of it. // We mess it up time after time, and something comes that we have to implement with short notice. So we try to force it on the organization, so we forget that we need to get the support of the co-workers.* Leader 4.

The propensity for change differs both among individuals and within the organization. According to the leaders, that could pose a challenge, since the support and needs differ between individuals. The motivational aspect could also vary between different actors, and some leaders claim that it is crucial to arouse curiosity among healthcare professionals. If the leaders are not motivated and do not believe that the change benefits them, implementation will not be successful. To increase healthcare professionals’ motivation and engagement, the value that will be created for the clinicians has to be made obvious, along with whether the AI system will support them in their daily work.

*It has to be beneficial for the clinics otherwise it’s meaningless so to speak. A big risk with AI is that you work and work with data and then algorithms emerge that are sort of obvious. Everyone can do this. It’s why it’s important to have clinical staff in the small agile teams, that there really is a clinical benefit, this actually improves it.* Leader 10.

#### Developing new strategies for internal and external collaboration

The healthcare leaders believed that there was a need for new forms of collaboration and communication within the county council, at both organizational and professional levels. Professionals need to interact with professions other than their own, thus enabling new teamwork and new knowledge. The challenge is for different groups to talk to each other, since they do not always have the same professional language. However, it was perceived that, when these kinds of team collaborations are successful, there will be benefits, such as automation of care processes that are currently handled by humans.

*To be successful in getting a person with expert knowledge in computer science to talk to a person with expert knowledge in integrity legislation, to a one who has expert knowledge in the clinical care of a patient. Even if all of them go to work with exactly the same objective, that one person or a few people can live a bit longer or feel a bit better, then it’s difficult to talk with each other because they use essentially different languages. They don’t know much about what knowledge the other has, so just getting that altogether.* Leader 2.

Leaders’ views the implementation of AI systems would require the involvement and collaboration of several departments in the county council across organizational boundaries, and with external actors. A perceived challenge was that half of the primary care units are owned by private care providers, where the county council has limited jurisdiction, which challenges the dissemination of common ways of working. Additionally, the organization in the county council and its boundaries might have to be reviewed to enable different professions to work together and interact on an everyday basis.

*The complexity in terms of for example apps is very, very, very much greater, we see that now. Besides there being this app, so perhaps the procurement department must be involved, the systems administration must definitely be involved, the knowledge department must be involved and the digitalization department, there are so many and the finance department of course and the communication department, the system is thus so complex.* Leader 9.

There was also consensus among the healthcare leaders that the county council should collaborate with companies in AI systems implementation and should not handle such processes on their own. An eco-system of actors working in AI systems implementation is required, who have shared goals for the joint work. The leaders expressed that companies must be supported and invited to collaborate within the county council’s organization at an early stage. In that way, pitfalls regarding legal or technical aspects can be discovered early in product development. Similar relations and dialogues are also needed with patients to succeed with implementation that is not primarily based on technical possibilities, but patients’ needs. Transparency is essential to patients’ awareness of AI systems’ functions and for the reliability in outcomes.

*This is born out of a management philosophy, which is based on the principle of not being able to command everything oneself, one has to be humble, perceptive about not being able to do it. One needs to invite others to be there and help with the solution.* Leader 16.

### Transformation of healthcare professions and healthcare practices

#### Managing new roles in care processes

The healthcare leaders described a need for new professions and professional roles in healthcare for AI systems implementation. All professional groups in today’s healthcare sector were expected to be affected by these changes, particularly the work unit managers responsible for daily work processes and the physicians accountable for the medical decisions. The leaders argued that the changes could challenge traditions, hierarchies, conventional professional roles and division of labour. There might be changes regarding the responsibilities for specific work tasks, changes in professional roles, a need for new professions that do not exist in today’s labour market and the AI systems might replace some work tasks and even professions. A change towards more combined positions at both the county council and a company or a university might also be a result of the development and implementation of AI systems. However, the leaders perceived that, for some healthcare professionals, these ideas are unthinkable, and it may take several years before these changes in roles and care processes become a reality in the healthcare sector.

*I think I will be seeing other professions in the healthcare services who have perhaps not received a healthcare education. It will be a culture shock, I think. It also concerns that you may perhaps not need to be medically trained, for sitting there and checking those yellow flags or whatever they are, or it could perhaps be another type of professional group. I think that it would actually be good. We have to start economizing with the competencies we now have and it’s difficult enough to manage.* Leader 15.

The acceptance of the AI systems may vary within and between professional groups, ages, and areas of specialized care. The leaders feared that the implementation of AI systems would change physicians’ knowledge base and that there would be a loss of knowledge that could be problematic in the long run. The leaders argued that younger, more recently graduated physicians would never be able to accumulate the experience-based knowledge to the extent that their older colleagues have done, as they will rely more on AI systems to support their decisions. Thus, on one hand, professional roles and self-images might be threatened when output from the AI systems is argued to be more valid than the recommendation by an experienced physician. However, on the other hand, physicians who do not “work with their hands” can utilize such output as decision support to complement their experience-based knowledge. Thus, it is important that healthcare professionals have trust in recommendations from the AI systems in clinical practice. If some healthcare professionals do not trust the AI systems and their output, there is a risk that they will not use them in clinical practice and continue to work in the way they are used to, resulting in two parallel systems. This might be problematic, both for the work environment and the healthcare professionals’ wellbeing. The leaders emphasized that this would represent a challenge for the implementation of AI systems in healthcare.

*We can’t add anything more today without taking something else away, I’d say it was impossible. // The level of burden is so high today so it’s difficult to see, it’s not sufficient to say that this will be of use to us in two years’ time.* Leader 20.

Implementing AI systems can change existing care processes and change the role of the patient. The leaders described that, in primary care, AI systems have the best potential to change existing work processes and make care more efficient, for example through an automatic AI-based triage for patients. The AI system could take the anamnesis, instead of the healthcare professionals, and do this when patients still are at home, so the healthcare professionals will not meet the patient unless the AI system has decided that it is necessary. The AI system can also autonomously discover something in a patient’s health status and suggest that the patient contact healthcare staff for follow-up. This use of AI systems could open up opportunities for more proactive and personalized care.

The leaders also described that the implementation of AI systems in practice could facilitate an altered patient role. The development that is taking place in the healthcare sector with, for instance, patient-reported data, enables and, in some cases, requires an active and committed patient that takes part in his or her care process. The leaders mentioned that there might be a need for patient support. Otherwise, there might be a risk that only patients with high digital literacy would be able to participate with valid data. The leaders described that AI systems could facilitate this development, by recommending self-care advice to patients or empowering them to make decisions. Still, there were concerns that not all patients would benefit from AI systems, due to variations in patients’ capabilities and literacy.

*We also deal with people who are ill, we must also have respect for that. Everyone will not be able to use these tools.* Leader 7.

#### Building trust for AI systems acceptance in clinical practice

A challenge and prerequisite for implementing AI systems in healthcare is that the technology meets expectations on quality to support the healthcare professionals in their practical work, such as having a solid evidence base, being thoroughly validated and meeting requirements for equality. It is important to have confidence in the validity of the data, the algorithms and their output. A key challenge pointed out was the need to have a sufficiently large population base, the “right” type of data and the right populations to build valid AI systems. For common conditions, where rich data exists to base AI algorithms, leaders believed the reliability would be high. For unusual conditions, there were concerns that there would be lower accuracy. Questions were also raised about how AI systems take aspects around equity and equality into account, such as gender and ethnicity. The leaders expressed concern that, due to these obstacles, in relation to certain unusual or complex conditions AI systems might not be suitable.

*Then there is a challenge with the new technology, whether it’s Ok to apply it. Because it’s people who are affected, people’s health and lives that are affected by the new technology. How can we guarantee that it delivers what it says it will deliver? It must be safe and reviewed, validated and evidence-based in order for us to be able to use it. If a bug is built in then the consequences can be enormous.* Leader 2.

Lack of confidence in the reliability of AI systems was also described and will place higher demands and requirements on their accuracy than on similar assessments made by humans. Thus, acceptance depends on confidence in AI systems as highly sensitive and that they can diagnose conditions at earlier stages than skilled healthcare professionals. The leaders perceived that the “black box” needs to be understood in order to be reliable, i.e. what the AI algorithms calculations are based on. Thus, reliance on the outputs from AI algorithms depends on reliance on the algorithm itself and the data used for its calculation.

*There are a number of inherent problems with AI. It’s a little black box. AI looks at all the data. AI is not often easy to explain, “oh, you’ve got a risk, that it passed the cut-off value for that person or patient”, no because it weighs up perhaps a hundred different dimensions in a mathematical model. AI models are often called a black box and there have been many attempts at opening that box. The clinics are a bit skeptical then when they are not able to, they just get a risk score, I would say.* Leader 10.

Big data sets are important for quality, but the leaders stated that too much information about a patient also could be problematic. There is a risk that information about a patient is available to healthcare professionals who should not have that information. The leaders believed that this could already be a problem today, but that it would be an increased risk in the future. This challenge needs to be handled as the amount of patient information increases, and as more healthcare professionals get access to such information when it’s being used in AI systems, regardless of the reason for the patient’s contact with the healthcare unit. Another challenge and prerequisite for implementing AI systems in healthcare is that the technology is user-friendly and create value for both healthcare professionals and patients. The leaders expected AI systems to be user-friendly, self-instructing, and easy to use, without requiring too much prior knowledge or training. In addition to being easy to use, the AI systems must also be time-saving and never time-consuming or dependent on the addition of yet more digital operative systems to work with. Using AI systems should, in some cases, be equated with having a second opinion from a colleague, when it comes to simplicity and time consumption.

*An easy way to receive this support is needed. One needs to ask a number of questions in order to receive the correct information. But it mustn’t be too complicated, and it mustn’t take time, then nothing will come of it.* Leader 4.

The leaders expected that AI systems would place the patients in focus and thereby contribute to more person-centred care. These expectations are based on a large amount of data on which AI algorithms are built, which leaders perceive will make it possible to individualize assessments and treatment options. AI systems would enable more person-centred and value-creating care for patients. AI systems could potentially contribute to making healthcare efficient without compromising quality. It was seen as an opportunity to meet future increasing needs for care among the citizens, combined with a reduced number of healthcare professionals. Smart and efficient AI systems used in investigations, assessments, and treatments can streamline care and allow more patients to receive care. Making healthcare efficient was also about the idea that AI systems should contribute to improved communication within and between caregivers for both public and private care. Using AI systems to follow up the given care and to evaluate the quality of care with other caregivers was highlighted, along with the risk that the increased efficiency provided by AI systems could result in a loss of essential values for healthcare and in impaired care.

*I think that automatization *via* AI would be a safe way and it would be perfect for the primary care services. It would have entailed that we have more hands, that we can meet the patients who need to be met and that we can meet more often and for longer periods and perhaps do more house calls and just be there where we are needed a little more and help these a bit more easily.* Leader 13.

## Discussion

The perspectives of the challenges described by leaders in the present study are an important contribution to improving knowledge regarding the determinants influencing the implementation of AI systems in healthcare. Our results showed that healthcare leaders perceived challenges to AI implementation concerning the handling of conditions external to the healthcare system, the building of internal capacity for strategic change management and the transformation of professional roles and practices. While implementation science has advanced the knowledge concerning determinants for successful implementation of digital technology in healthcare [53], our study is one of the few that have investigated leaders’ perceptions of the implementation of AI systems in healthcare. Our findings demonstrate that the leaders concerns do not lie so much with the specific technological nuances of AI, but with the more general factors relating to how such AI systems can be channeled into routine service organization, regulation and practice delivery. These findings demonstrate the breadth of concerns that leaders perceive are important for the successful application of AI systems and therefore suggest areas for further advancements in research and practice. However, the findings also demonstrate a potential risk that, even in a county council where there is a high level of investment and strategic support for AI systems, there is a lack of technical expertise and awareness of AI specific challenges that might be encountered. This could cause challenges to the collaboration between the developers of AI systems and healthcare leaders if there is a cognitive dissonance about the nature and scope of the problem they are seeking to address, and the practical and technical details of both AI systems and healthcare operational issues [7]. This suggests the need for people who are conversant in languages of both stakeholder groups maybe necessary to facilitate communication and collaboration across professional boundaries [54]. Importantly, these findings demonstrate that addressing the technological challenges of AI alone is unlikely to be sufficient to support their adoption into healthcare services, and AI developers are likely to need to collaborate with those with expertise in healthcare implementation and improvement scientists in order to address the wider systems issues that this study has identified.

### Conditions external to the healthcare system

The healthcare leaders perceived challenges resulting from external conditions and circumstances, such as ambiguities in existing laws and sharing data between organizations. The external conditions highlighted in our study resonate with the outer setting in the implementation framework CFIR [37], which is described in terms of governmental and other bodies that exercise control, with the help of policies and incentives that influence readiness to implement innovations in practice. These challenges described in our study resulted in uncertainties concerning responsibilities in relation to the development and implementation of AI systems and what one was allowed to do, giving rise to legal and ethical considerations. The external conditions and circumstances were recognized by the leaders as having considerable impact on the possibility of implementing AI systems in practice although they recognized that these were beyond their direct influence. This suggests that, when it comes to the implementation of AI systems, the influence of individual leaders is largely restricted and bounded. Healthcare leaders in our study perceived that policy and regulation cannot keep up with the national interest in implementing AI systems in healthcare. Here, concerted and unified national authority initiatives are required according to the leaders. Despite the fact that the introduction of AI systems in healthcare appears to be inevitable, the consideration of existing regulatory and ethical mechanisms appears to be slow [16, 18]. Additionally, another challenge attributable to the setting was the lack of to increase the competence and expertise among professionals in AI systems, which could be a potential barrier to the implementation of AI in practice. The leaders reflected on the need for future higher education programs to provide healthcare professionals with better knowledge of AI systems and its use in practice. Although digital literacy is described as important for healthcare professionals [55, 56], higher education faces many challenges in meeting emerging requirements and demands of society and healthcare.

### Capacity for strategic change management

The healthcare leaders addressed the fact that the healthcare system’s internal capacity for strategic change management is a hugh challenge, but at the same time of great importance for successful and sustainable implementation of AI systems in the county council. The leaders highlighted the need to create an infrastructure and joint venture, with common structures and processes for the promotion of the capability to work with implementation strategies of AI systems at a regional level. This was needed to obtain a lasting improvement throughout the organization and to meet organizational goals, objectives, and missions. Thus, this highlights that the implementation of change within an organization is a complex process that does not solely depend on individual healthcare professionals’ change responses [57]. We need to focus on factors such as organisational capacity, climate, culture and leadership, which are common factors within the “inner context” in CFIR [37]. The capacity to put the innovations into practice consists of activities related to maintaining a functioning organization and delivery system [58]. Implementation research has most often focused on implementation of various individual, evidence-based practices, typically (digitally) health interventions [59]. However, AI implementation represents a more substantial and more disruptive form of change than typically involved in implementing new practices in healthcare [60]. Although there are likely many similarities between AI systems and other new digital technologies implemented in healthcare, there may also be important differences. For example, our results and other AI research has acknowledged that the lack of transparency (i.e. the “black box” problem) might yield resistance to some AI systems [61]. This problem is probably less apparent when implementing various evidence-based practices based on empirical research conducted according to well-established principles to be trustworthy [62]. Ethical and trust issues were also highlighted in our study as playing a more prominent role in AI implementation, perhaps more prominently than in “traditional” implementation of evidence-based practices. There might thus be AI-specific characteristics that are not really part of existing frameworks and models currently used in implementation science.

### Transformation of healthcare professions and healthcare practice

The healthcare leaders perceived that the use of AI in practice could transform professional roles and practices and this could be an implementation challenge. They reflected on how the implementation of AI systems would potentially impact provider-patient relationships and how the shifts in professional roles and responsibilities in the service system could potentially lead to changes in clinical processes of care. The leaders’ concerns related to the compatibility of new ways of working with existing practice, which is an important innovation characteristic highlighted in the Diffusion of Innovation theory [63]. According to the theory, compatibility with existing values and past experiences facilitates implementation. The leaders in our study also argued that it was important to see the value of AI systems for both professionals and service-users. Unless the benefits of using AI systems are observable healthcare professionals will be reluctant to drive the implementation forward. The importance of observability for adoption of innovations is also addressed in the Diffusion of Innovation theory [63], being the degree to which the results of an innovation are visible to the users. The leaders in our study conveyed the importance for healthcare professionals of having trust and confidence in the use of AI systems. They discussed uncertainties regarding accountability and liability in situations where AI systems impacts directly or indirectly on human healthcare, and how ambiguity and uncertainty about AI systems could lead to healthcare workers having a lack of trust in the technology. Trust in relation to AI systems is well reflected on as a challenge in research in healthcare [30, 41, 64–66]. The leaders also perceived that the expectations of patient-centeredness and usability (efficacy and usefulness) for service users could be a potential challenge in connection with AI implementation. Their concerns are echoed in a review by Buchanan et al. [67], in which it was observed that the use of AI systems could serve to weaken the person-centred relationships between healthcare professionals and patients.

In summary, the expectations for AI in healthcare are high in society and the technological impetus is strong. A lack of “translation” of the technology is in some ways part of the initial difficulties of implementing AI, because implementation strategies still need to be developed that might facilitate testing and clinical use of AI to demonstrate its value in regular healthcare practice. Our results relate well to the implementation science literature, identifying implementation challenges attributable to both external and internal conditions and circumstances [37, 68, 69] and the characteristics of the innovation [37, 63]. However, the leaders in our study also pointed out the importance of establishing an infrastructure and common strategies for change management on the system level in healthcare. Thus, introducing AI systems and the required changes in healthcare practice should not only be dependent on early adopters at the particular units. This resonates with the Theory of Organizational Readiness for Change [70], which emphasizes the importance of an organization being both willing *and* able to implement an innovation [71]. The theory posits that, although organizational willingness is one of the factors that may facilitate the introduction of an innovation into practice, both the organization’s general capacities and its innovation-specific capacities for adoption and sustained use of an innovation are key to all phases in the implementation process [71].

### Methodological considerations

In qualitative research, the concepts credibility, dependability, and transferability are used to describe different aspects of trustworthiness [72]. *Credibility* was strengthened by the purposeful sample of participants with various experiences and a crucial role in any implementation process. It is considered of great relevance to investigate the challenges that leaders in the county council expressed concerning the implementation of various AI systems in healthcare, albeit the preparation for implementing AI systems is a current issue in many Swedish county councils. Furthermore, the research team members’ familiarity with the methodology, together with their complementary knowledge and backgrounds enabled a more nuanced and profound, in-depth analysis of the empirical material and was another strength of the study.

*Dependability* was strengthened by using an interview guide to ensure that the same opening questions were put to all participants and that they were encouraged to talk openly. Because this study took place during the COVID-19 pandemic, the interviews were performed either at a distance, using the Microsoft Teams application, or face-to-face, the variation might be a limitation. However, according to Archibald et al. [73], distance interviewing with videoconferencing services, such as Microsoft Teams, could be beneficial and even preferred. Based on the knowledge gap regarding implementation of AI systems in healthcare, the authors chose to use an inductive qualitative approach to the exploration of healthcare leaders’ perceptions of implementation challenges. It might be that the implementation of AI systems largely aligns with the implementation of other digital technologies or techniques in healthcare. A strength of our study is that it focuses on perceptions on AI systems in general regardless of the type of AI algorithm or the context or area of application. However, one potential limitation of this approach is the possibility that more specific AI systems and or areas of applications may become associated with somewhat different challenges. Further studies specifying such boundaries will provide more specific answers but will probably also require the investigation be conducted in connection with the actual implementation of a specific AI systems and based on participants' experiences of having participated in the implementation process. With this in mind, we encourage future research to take this into account when deciding upon study designs.

*Transferability* was strengthened by a rich presentation of the results along with appropriate quotations. However, a limitation could be that all healthcare leaders work in the same county council, so transferability to other county councils must be considered with caution. In addition, an important contextual factor that might have an impact on whether, and how, the findings observed in this study will occur in other settings as well, concerns the nature of, and approach to, AI implementation. AI could be considered a rather broad concept, and while we adopted a broad and general approach to AI systems in order to understand healthcare leader’s perceptions, we would, perhaps, expect that more specific AI systems and or areas of applications become associated with different challenges. Taken together, these are aspects that may affect the possibilities for our results to be portable or transferred to other contexts. We thus suggest that the perceptions of healthcare leaders in other empirical contexts and the involvement of both more specific and broader AI systems are utilized in the study designs of future research.

## Conclusion

In conclusion, the healthcare leaders highlighted several implementation challenges in relation to AI within the healthcare system and beyond the healthcare organization. The challenges comprised conditions external to the healthcare system, internal capacity for strategic change management, and transformation of healthcare professions and healthcare practice. Based on our findings, there is a need to see the implementation of AI system in healthcare as a changing learning process at all organizational levels, necessitating a healthcare system that applies more nuanced systems thinking. It is crucial to involve and collaborate with stakeholders and users inside the regional healthcare system itself and other actors outside the organization in order to succeed in developing and applying system thinking on implementation of AI. Given that the preparation for implementing AI systems is a current and shared issue in many (Swedish) county councils and other countries, and that our study is limited to one specific county council context, we encourage future studies in other contexts, in order to corroborate the findings.

## Data Availability

Empirical material generated and/or analyzed during the current study are not publicly available, but are available from the corresponding author on reasonable request.
